# Estimation of the relationship between the persistent decrease of the suicide rate and the changes in sociodemographic composition in Hungary between 1990 and 2011

**DOI:** 10.1371/journal.pone.0241314

**Published:** 2020-10-23

**Authors:** Lajos Balint, Katalin Fuzer, Xenia Gonda, Peter Dome

**Affiliations:** 1 Demographic Research Institute of the Hungarian Central Statistical Office, Budapest, Hungary; 2 Department of Sociology, University of Pécs, Pécs, Hungary; 3 Department of Psychiatry and Psychotherapy, Semmelweis University, Faculty of Medicine, Budapest, Hungary; 4 Nyiro Gyula National Institute of Psychiatry and Addictions, Budapest, Hungary; 5 NAP-2-SE New Antidepressant Target Research Group, Hungarian Brain Research Program, Semmelweis University, Budapest, Hungary; Universita degli Studi Europea di Roma, ITALY

## Abstract

From the end of the 1980s, the Hungarian suicide rate, which had been until that point the highest in the world for decades, has decreased drastically. The reason behind this decrease was probably due to the changes in numerous and often interlinked risk factors. Studies on this topic have mostly ignored to interrogate to what extent the *change* of the population composition, for example the improvement of educational level, contributed to the decrease of the rate in the given period? Our aim was to assess the contribution of changes in some sociodemographic factors to the decrease of the suicide rate in Hungary. During the analysis, data from 1990 were compared with data from 2011. For the statistical calculations, the method of “Standardization and Decomposition (SDA)” was used, which according to our best knowledge, has not yet been applied in Hungarian suicide studies. The results show that the improvement of educational level helped to decrease the rate for men by about a third, while for women only by about a tenth. However, the benefit of the improvement in educational attainment during the period investigated was significantly offset by the changes primarily in marital status (the ratios of unmarried and divorced subjects increased for both genders) and in age distribution (the ratio of the elderly persons increased for both genders). The results of our study emphasise the inverse relationship between suicide and educational level and support the hypothesis that we can regard educational policy as indirect health policy.

## Introduction

**Fig 1:**
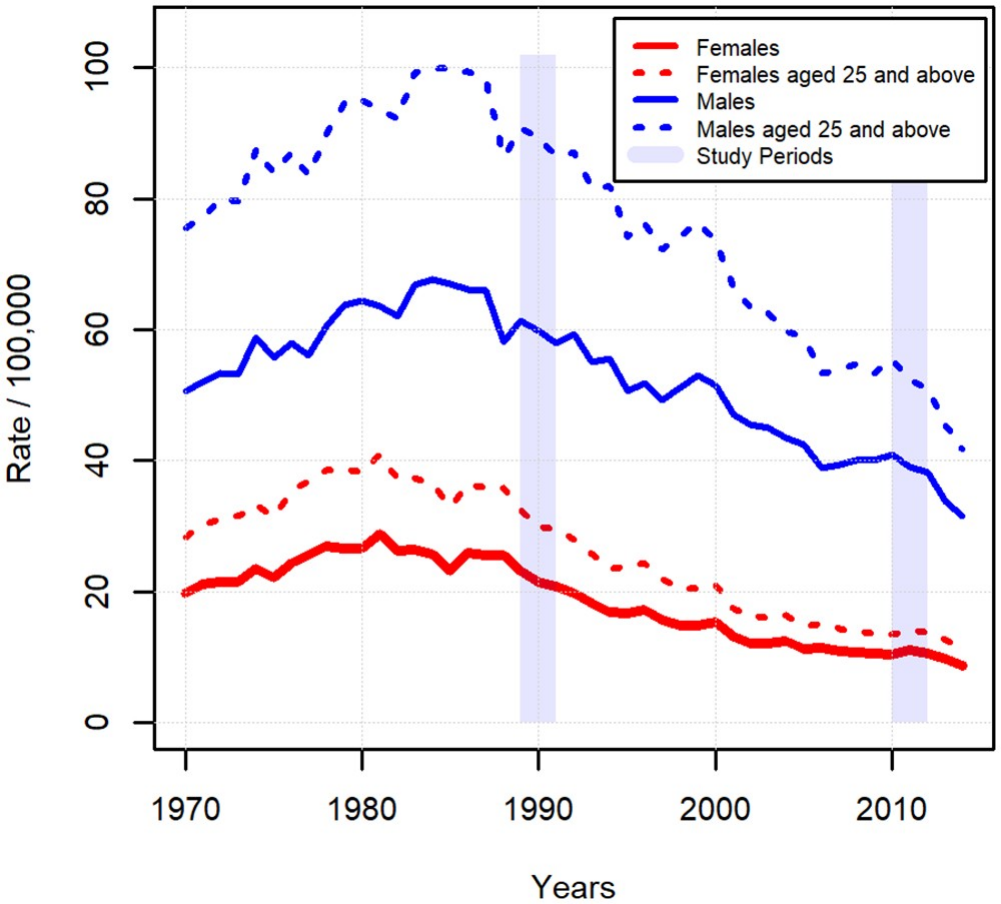
Hungarian raw suicide rate (/100,000 capita) between 1970 and 2014 for the whole population and population of 25 years of age and above, by genders.

The period between 1960 and 2000 saw the Hungarian suicide rate as the highest in the world [1]. In the middle of the 90s the suicide rate of males in some countries of the post-communist region reached an even higher level than that which had ever been experienced in Hungary, while according to official data no female population of any European country was capable of surpassing the Hungarian rates of the 80s [2, 3]. In Hungary, the rate was at its highest in the 80s (for women at the end of the 70s and beginning of the 80s, while for men at the end of the decade) and then its lasting and significant decrease for the whole population started at the end of the 80s. The decrease temporarily stopped for a few years, following the financial crisis of 2007/8 but continued thereafter (Fig 1) [1, 4, 5]. Interestingly enough, the improvement of suicide rate started in a period of the 80s when in Hungary, and also in the majority of post-communist countries, the overall mortality deteriorated further [6–8]. Although in today’s Hungary the suicide rate is far lower than ever before in the previous four decades, following the millennium the Hungarian suicide rates for both genders were still double that of the EU average [2]. It is worth mentioning that contrary to Hungary, where regarding the whole population the beginning of the decrease in suicide rate concurred more or less at the time of the regime change, in other post-communist countries (e.g. the Baltic states) this decrease only happened years after the political and economic transition, and there are also countries where there is still no lasting decrease to be found (e.g. in the case of Poland and Romania) [1, 9]. Taking into account that in the post-communist countries the regime change did not result in a universal improvement of the suicidal scene, and also that the suicide rate for Hungarian women started to decrease years before the regime change, it can be stated with great certainty that favourable political changes (i.e. the transition from the socialist one-party dictatorship to a democratic market economy) and the concomitant social optimism can only partly explain the decrease of Hungarian suicide rate started at the time of the regime change [1, 4].

There is no consensus on the reasons behind the decrease of suicide rate that has been going on since the end of the 80s as the results of investigations using different methodologies on data from various periods are quite inconsistent. Ever since the millennium several investigations have been conducted, focusing on Eastern Europe (including Hungary), or concentrating only on Hungary, to examine the complex relationships between social and economic changes, health behaviour, certain markers of the healthcare system and suicide [7, 8, 10–14]. These investigations on the connections between certain indicators of the economy (e.g. GDP, unemployment), marital status, divorce rate, alcohol consumption and smoking, markers of psychiatric care (e.g. antidepressant sales) and the suicide rate have often achieved contradictory results. Since in our study the main explanatory variables were marital status, educational attainment, gender and age, we summarise briefly the results achieved in connection with them so far.

The role of *educational attainment* in the determination of suicidal behaviour was already proposed in the 19th century by such classical authors of suicidology as Durkheim and Morselli. Both of them concluded that suicide prevails among subjects with higher education [13, 15, 16]. However, the results only of a few recent studies are in consonance with the views of Durkheim and Morselli [17, 18], the overwhelming majority of contemporary findings suggest that the risk of suicidal behaviour is negatively correlated with higher educational attainment [see discussed in the Introduction of 13, 19, 20]. Of note, educational attainment probably has no direct effect on suicide risk but it has links to various other factors that may influence suicide risk more directly such as alcohol and tobacco consumption, carrier opportunities and personal income or access to mental health care [21, 22]. Furthermore higher educated subjects have more opportunity to be members of tight-knit prosocial groups which may help them to marry and to get jobs [23]. Some studies have found that the inverse relationship between educational attainment and suicide risk is less marked or even the opposite among females [13, 24]. Explanations of this phenomenon may be found in the literature and mainly concentrated around the fact that the same level of education provides greater social activities and career opportunities for men than for women [13, 24]. In Hungary, only a few studies investigated the association between educational attainment and suicidal behaviour. Among these, two psychological autopsy studies have provided contradictory results [25, 26]. Furthermore, a previous study of ours found that higher educational attainment is significantly associated with decreased risks of suicide and this effect was stronger among males [13].

We may state that there is a consensus about the existence of an association between *marital status* and suicide risk. This association was already described by Morselli and Durkheim and has been confirmed by a large number of later studies. According to the results of historical and contemporary studies, non-married (e.g. divorced, widowed, unmarried) statuses are associated with a higher suicide risk than married status [13, 19, 27]. Several theories may explain this finding: for example, living in marriage gives social and economic support, spouses may also encourage each other to live a healthy lifestyle that is protective against depression. Furthermore, those living in marriage may differ in several ways from those who are divorced; for instance they may be healthier than divorced individuals since when a spouse becomes chronically ill, rising personal and economic loads may cause couples to divorce, and the ill spouse may have less chance to remarry. This so called “matrimonial selection bias” may be another mediator factor between marital status and suicide [27, 28]. Intriguingly, among non-married statuses, divorced status was generally found to be associated with the highest risk of suicide [13, 24, 27]. Similarly to the association between educational attainment and suicide risk, it might be said that the effect of marital status on suicide risk is gender-specific: the majority of findings suggest that the protective effect of marriage is more pronounced among males, especially above 65 years of age (for instance, according to the results of a meta-analysis, there is no association between marital status and suicide risk in females above 65 years of age) [13, 24, 27]. Various theories were put forward to explain this phenomenon, for instance greater role inflexibility and fewer alternative intimate relationships—i.e. weaker social network outside the family—are more characteristic of males than females. Furthermore, as a consequence of divorce, men more frequently lose their close relationship with children and their home than females [13, 24]. In a previous study of ours, we demonstrated in datasets from 1980, 1990, 2001 and 2011 that, in line with the majority of results from other countries, non-married statuses pose an extra risk of suicide in Hungary, and that the effect of marital status was stronger in men [13]. Another Hungarian study found the highest suicide rates among divorced and widowed males and females in all age groups in 1980 and 2010 [1].

Almost in all countries of the world (with the notable exception of China) suicide rate of males is higher than that of females [29]. *Gender differences* in suicide risk in Hungary are in line with the previously mentioned global male-to-female preponderance (the male-to-female suicide ratios in Hungary were 2.53 in 1983 and 3.93 in 2010) [1].

With regard to *age*, suicide rates are highest in elderly persons for both males and females in almost all regions of the globe [30]. In accordance with this general finding, in Hungary suicide rates for women were increased with increasing age both in 1980 and 2010 (in the same years the same trend was observable for men, but curves were rather bimodal with a flattening in the age-group 60–69) [1].

The aim of the current study was to assess to what extent changes in the composition of the population during the two decades after the political transition may be responsible for the decrease of suicide rate in Hungary.

## Materials and methods

In the analysis unlinked cross-sectional data were used. The data were categorised by gender, age groups of five years (i.e. 25–29, 30–34 ….. 85-x), marital status (married, unmarried, divorced, and widowed) and educational attainment. For educational attainment, we strived for the most detailed categorisation possible because using too wide groups has the dangerous effect of obscuring the real differences between the groups. Due to the fact that our analysis covers the whole age segment of adulthood, we attempted to select groups which contained risk population for both period. In our analysis, it was possible to separate four groups based on educational attainment. Those with under 8 years of primary education constituted the group with the lowest educational attainment. The introduction of vocational education happened in the beginning of the 60s (in 1990 those with vocational education were at most in the beginning of their forties). Because their age-specific suicide rates showed similarity to that of those who completed primary education only, we decided to merge these two groups. Those with higher education were separated into two groups: those who have graduated from a high-school and those who have graduated from university or college. The data for the population reflect the situation at the time when the censuses were conducted. The data for mortality by suicide (ICD9: E950.0-E959.0, ICD10: X60-X84) came from the Demographic/Vital Register ("DEMO") of the Hungarian Central Statistical Office ("HCSO"). This electronic database consists routinely collected data. Our data from the "DEMO" do not contain any personal sensitive information, such as name, social security number etc. This statement is also true for our data derived from the Census database. We used grouped (and not individual level) data. Census data were similarly tabulated—according to sex, age groups, marital status and educational attainment—as mortality data from the "DEMO". The ICD codes for suicide were chosen in accordance with the practice of scientific literature [10, 12]. Similarly to other studies investigating comparable questions we included subjects beyond the age of 25 years [21, 24] because the course of education is largely over by this age and the highest educational attainment achieved changes very little during the later course of life. In order to moderate the uncertainty related to the low number of cases for some groups, mortality cases from three consecutive years (1989–91 and 2010–2012) were taken into account. Accordingly, three times the size of the population was taken as risk population, and it was assumed that in the years preceding and succeeding the census the population structure was equal to those of the censuses.

In the second period (2010–2012) for 6.5% of all deaths (n = 455) the educational attainment was unknown. The missing values were replaced with the help of multivariate imputations by chained equations [31].

The difference between the overall rates in the two periods was divided into two additive factors by the method of standardization and decomposition analysis (SDA) which is widely used in demographics: (1) the *rate effect* attributed to the difference in specific death rates, as well as (2) the *factor component* effect attributed to the difference in specific factor variable structure (e.g. age structure between two populations). The former is the „real” rate difference; the latter is the rate difference due to confounding effects [32–34]. The standardization and decomposition analysis was carried out with the help of the software DECOMP which is free to download from the site http://www.wright.edu/~jichuan.wang. The SDA has numerous advantages: it is based on algebraic calculation and as such, there are no restrictions on the specification of the relation (e.g. linearity), the nature of the variables (e.g. randomness), the form of variable distribution (e.g. normality) and the independence of the observations, as the base assumption of standard statistical analyses [34].

In the analysis, the average difference of suicide rate in 1990 and 2011 was divided into 5 factors: the first factor expresses the effect of the composition of the age structure of the two populations; the second factor expresses the effect of gender differences; the third the effect of educational attainment, while the fourth expresses the effect of the composition of marital status. The rate effect is attributed to the different factor-specific rates. For the decompositions separated for genders, the number of factors was one less.

## Results

### Change of population composition

Between 1990 and 2011, the population aged 25 and above increased by almost half a million (however, the population under 25 years of age decreased far more significantly, by more than 900 thousand) (Table 1). The change of the age structure of the population was characterised by the decrease of people under 50 years of age and the increase of those over 50 years of age. Owing to the improvement of elderly mortality, there was a great increase in the number of people above 80 years of age (Table 1). This change in the age structure was similar for both men and women.

**Table 1:** Number of subjects aged 25 years and above by educational attainment, age, marital status and gender in 1990 and 2011.

Demographic groups	Males (n, %)		Females (n, %)		Total (n, %)	
	1990	2011	1990	2011	1990	2011
**Age-groups**						
25–29	31367 (9,9)	310238 (9,2)	306619 (8,4)	301063 (7,7)	620290 (9,1)	611301 (8,4)
30–34	389033 (12,3)	385903 (11,5)	385382 (10,6)	379414 (9,7)	774415 (11,4)	765317 (10,5)
35–39	423099 (13,4)	412285 (12,3)	424242 (11,6)	403311 (10,3)	847341 (12,5)	815596 (11,2)
40–44	355443 (11,3)	358261 (10,7)	361232 (9,9)	356260 (9,1)	716675 (10,5)	714521 (9,8)
45–49	328144 (10,4)	297246 (8,9)	346810 (9,5)	304426 (7,8)	674954 (9,9)	601672 (8,3)
50–54	277582 (8,8)	315132 (9,4)	320119 (8,8)	342439 (8,7)	597701 (8,8)	657571 (9,0)
55–59	279920 (8,9)	364059 (10,9)	327623 (9,0)	417052 (10,6)	607543 (8,9)	781111 (10,7)
60–64	259856 (8,2)	293440 (8,7)	326068 (8,9)	360551 (9,2)	585924 (8,6)	653991 (9,7)
65–69	221183 (7,0)	221918 (6,6)	308485 (8,5)	301053 (7,7)	529668 (7,8)	522971 (7,2)
70–74	106513 (3,4)	162896 (4,9)	161269 (4,4)	260948 (6,6)	267782 (3,9)	423844 (5,8)
75–79	117075 (3,7)	115657 (3,4)	199509 (5,5)	215416 (5,5)	316584 (4,7)	331073 (4,5)
80–84	57685 (1,8)	73546 (2,2)	114744 (3,1)	160669 (4,1)	172429 (2,5)	234215 (3,2)
85-x	25008 (0,8)	43539 (1,3)	62451 (1,7)	121478 (3,1)	87459 (1,3)	165017 (2,3)
	*χ*^2^ = 29,744, *df* = 12, *P* < 0.001		*χ*^2^ = 57,232, *df* = 12, *P* < 0.001		*χ*^2^ = 85785, *df* = 12, *P* < 0.001	
**Educational attainment**						
Less than 8 years	631997 (20,0)	103184 (3.1)	1105989 (30.3)	280883 (7.2)	1737986 (25.6)	384067 (5.3)
Vocational school/primary school (8–11 yr)	1631500 (51.7)	1752514 (52.4)	1522191 (41.8)	1660274 (42.3)	3153691 (46.4)	3412788 (47.0)
Graduated from secondary-school (12–14 yr)	520014 (16.5)	879344 (26.3)	699454 (19.2)	1209603 (30.8)	1219468 (17.9)	2088947 (28.7)
Graduated from university/college (15≤ yr)	370701 (11.8)	609078 (18.2)	316919 (8.7)	773320 (19.7)	687620 (10.1)	1382398 (19.0)
	*χ*^2^ = 1321700, *df* = 3, *P* < 0.001		*χ*^2^ = 530060, *df* = 3, *P* < 0.001		*χ*^2^ = 815050, *df* = 3, *P* < 0.001	
**Marital status**						
Married	2423391 (76,8)	1871332 (55,8)	2321788 (63,7)	1874485 (47,8)	4745179 (69,8)	3745817 (51,5)
Unmarried	334263 (10,6)	929590 (27,7)	201551 (5,5)	649676 (16,6)	535814 (7,9)	1579266 (21,7)
Divorced	245993 (7,8)	401135 (12,0)	348630 (9,6)	577624 (14,7)	594623 (8,7)	978759 (13,4)
Widowed	150565 (4,8)	152063 (4,5)	772584 (21,2)	822295 (21,0)	923149 (13,6)	974358 (13,4)
	*χ*^2^ = 382,810, *df* = 2, *P* < 0.001		*χ*^2^ = 331,890, df = 2, *P* < 0.001		*χ*^2^ = 712,060, *df* = 2, *P* < 0.001	
** Total**	3154212 (100,0)	3354120 (100,0)	3644553 (100,0)	3924080 (100,0)	6798765 (100,0)	7278200 (100,0)

Source: HCSO Census data 1990, 2011, own calculation

The distribution by educational level in Eastern European societies was quite skewed in the last days of the state-socialist era. The proportion of lower educated was high and the proportion of highly educated people was extremely low compared to Western societies. In 1990, about a third of the female population (30.3%) and a fifth of the male population (20.0%) had less than 8 completed years of primary education. With the passing away of the older generations, the entry of new generations saw the number of those with less than 8 completed years of education decrease drastically: in 2011, 3.1% of the males and 7.2% of the females belonged to this group (Table 1). After the regime change, the ratio of those who have graduated from a secondary-school increased significantly, by 9.8% for men (from 16.5% to 26.3%), and a bit more significantly for women (from 19.2% to 30.8%) (Table 1). As a result of the expansion of higher education, the ratio of those who have graduated from university or college increased from 11.8% to 18.2% for men and from 8.7% to 19.7% for women (Table 1). Consistent with the experience of Western European countries, women became more educated than men. In 2011, the ratio of women who have graduated from a secondary-school or from university/college surpassed that of men (50.5% and 44.4%, respectively) while corresponding proportions were practically equal two decades before (27.9% and 28.2%, respectively) (Table 1). During the period investigated the proportion of those who were members of the vocational school/primary school group barely changed (Table 1).

In the period investigated in our study, the population composition by marital status changed significantly as well. The ratio of married people decreased dramatically between 1990 and 2011 (from 76.8% to 55.8% for men; from 63.7% to 47.8% for women). In the same period, the proportion of some groups with high suicide risk have increased. Accordingly, the proportion of unmarried increased from 10.6% to 27.7% for men and from 5.5% to 16.6% for women, and a similar, but somewhat smaller increases was also observable in the proportion of divorced subjects (from 7.8% to 12.0% for men; from 9.6% to 14.7% for women). At the same time, the ratio of widows/widowers did not change substantially for either gender (Table 1).

To summarise, in the time period under investigation, the population composition changed in a way that both the ratios of groups at greater risk (e.g. unmarried and the elderly) and at lower risk (the higher educated) of suicide have increased.

### The change of raw suicide rates according to educational attainment, marital status and age

Before discussing results, we would like to highlight that the Hungarian suicide data are considered reliable [20]. In Hungary the cause of death is determined exclusively by doctors (working in hospitals or GPs and/or pathologists). In case an extraordinary death is suspected (suicide, murder, accident, etc.) forensic autopsy is required according to the law [12]. The most common form of suicide was hanging (56.4% of all cases in 1990, 64.6% in 2010), and this form of suicide is harder to misclassify as non-suicide death than other forms of suicide (e.g. various poisonings) [35, 36]. In some countries, it was supposedly common to misclassify certain types of suicides as „deaths of undetermined intent”, in Hungary however, the number of these was negligible [10].

During the first period investigated (1989–91) 11840 (8472 by men; 3368 by women) suicides were committed in the population of subjects aged 25 and above, while the corresponding number during the second period investigated (2010–12) was 6956 (5342 by men; 1614 by women). The raw suicide rate for Hungarian men aged 25 and above decreased by about forty percent (from 89.5/100 000/year to 53.1/100 000/year), for women by more than 50 percent (from 30.8/100 000/year to 13.7/100 000/year). Due to the more significant relative improvement of female rates, the rate of men was three times (in 1990) and almost four times (by 2011) greater than that of the females (Table 2).

**Table 2:** Raw suicide rates by educational attainment, marital status and gender in the population aged 25 and above, in 1990 and 2011 (per 100,000 capita).

Groups	Males		Females		Total	
	1990	2011	1990	2011	1990	2011
**Educational attainment**						
Less than 8 years	134,97	100,79	41,53	23,38	75,51	44,18
Vocational school/primary school	99,71	70,54	29,34	17,59	65,76	44,86
Graduated from secondary-school	45.70	33.59	23.16	10.33	32.77	20.12
Graduated from university/college	28.41	22.55	17.25	7.16	23.27	13.94
**Marital status**						
Married	67,70	39,85	19,58	8,91	44,16	24,36
Unmarried	114,98	43,93	30,76	9,08	83,30	29,59
Divorced	178,87	104,29	49,53	22,91	103,03	56,26
Widowed	238,44	136,79	56,09	21,85	85,83	39,79
**Total**	89,51	53,07	30,80	13,71	58,04	31,85

The relationship between educational attainment and suicide mortality proved to work out as expected: with the increase of educational attainment, the frequency of completed suicides decreased. The rate differences between the groups, as it is shown by the absolute and relative rate differences between the most and lowest educated, were greater for men than for women (Table 2). The rates standardized with the age distribution of the European population showed similar results (these results are not shown here).

The married group had the lowest level of suicide mortality, they were followed by the unmarried, and the difference between their raw rates decreased to a minimal by 2011, possibly due to the fact that many members of the latter group in fact live in a relationship and thus benefit from the advantages of social support. In both genders, the divorced and the widowed had the highest tendency towards suicide. The absolute and relative difference between married and no longer married (i.e. divorced or widowed) was also greater for men than for women (Table 2).

**Fig 2:**
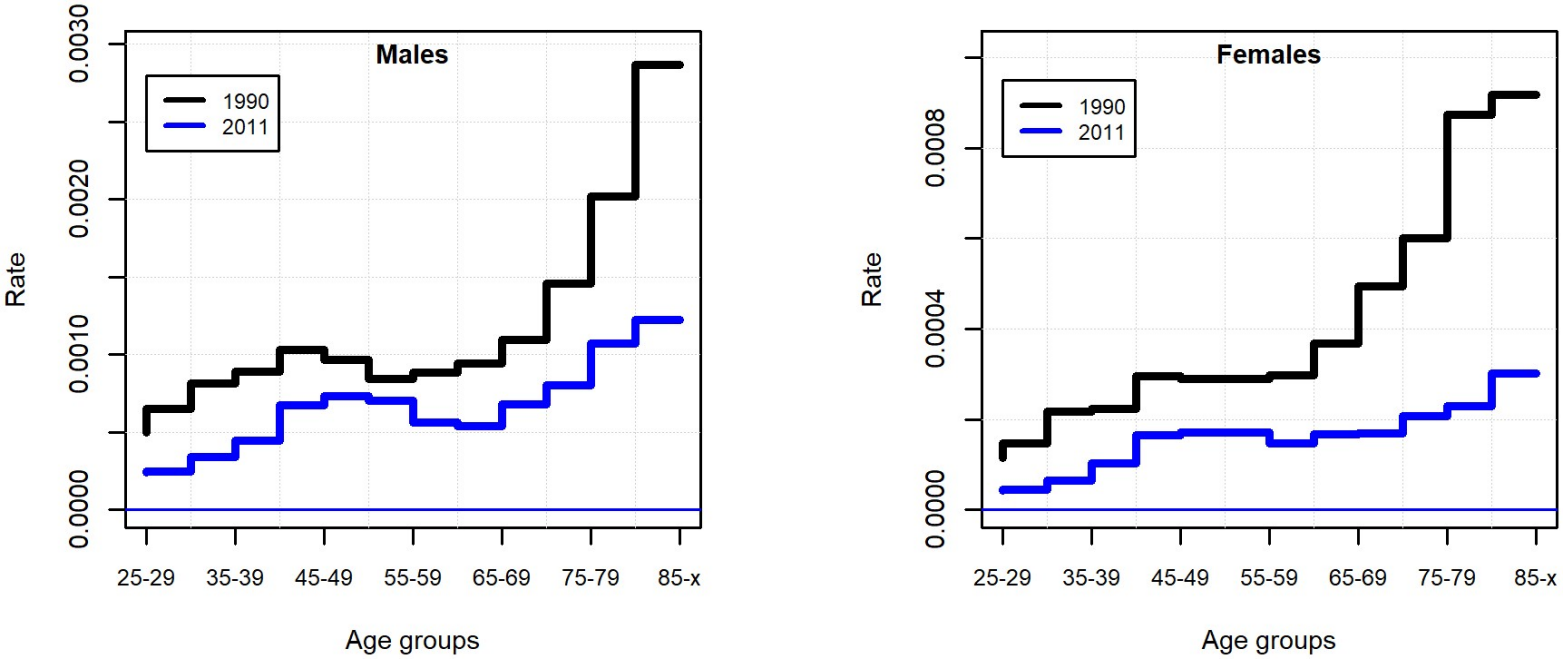
Suicide rate by age groups and genders in 1990 and 2011, for the population of 25 years of age and above.

In the period investigated, the suicide rate decreased in all age groups, the most considerable decrease happened for the oldest old. In the beginning of the 90s, the pattern of suicide by age showed a modest bimodal distribution for males, while the newer data show rather a suicide mortality increasing in a monotonic manner with age (Fig 2). Apart from the elderly, the increased suicide risk for males in economically active age is characteristic of Eastern European countries, which is supposed to be linked to alcohol consumption and economic traumas [1, 37].

**Fig 3:**
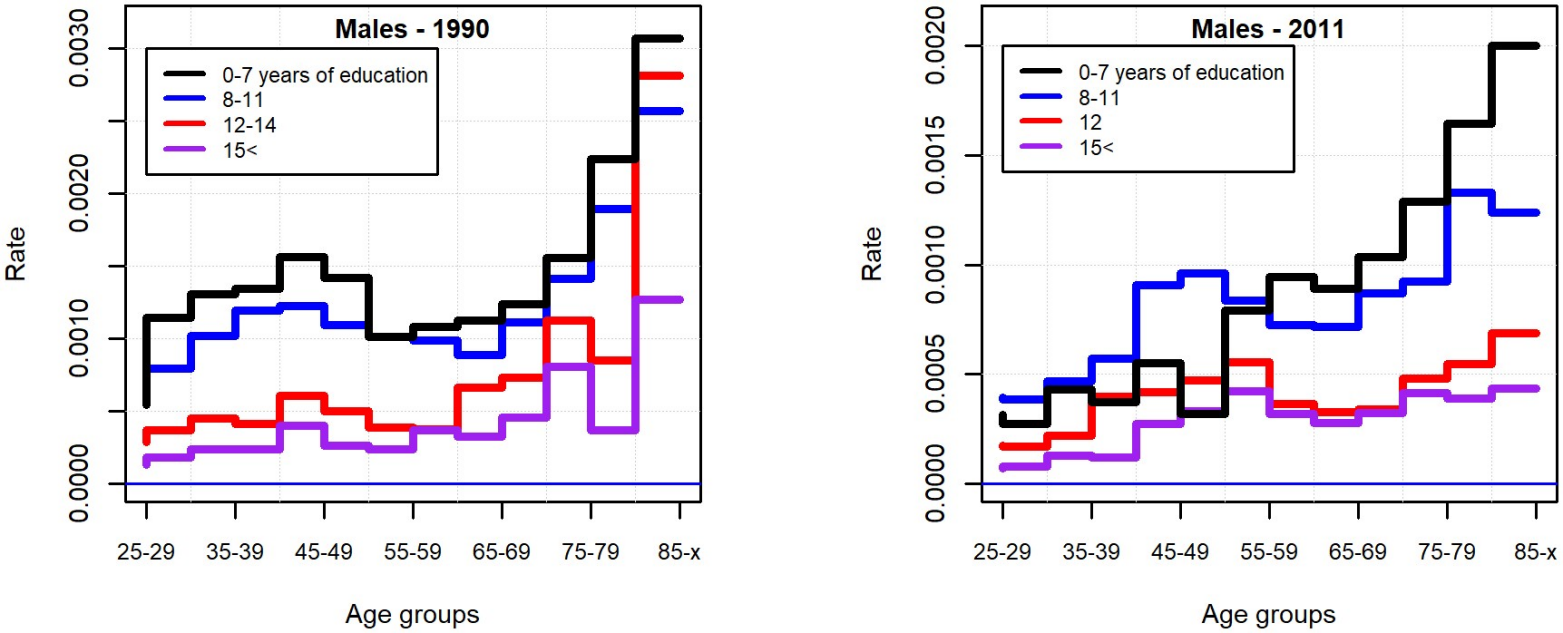
Suicide rate by educational attainment in 1990 and 2011 (males 25 years of age and above).

**Fig 4:**
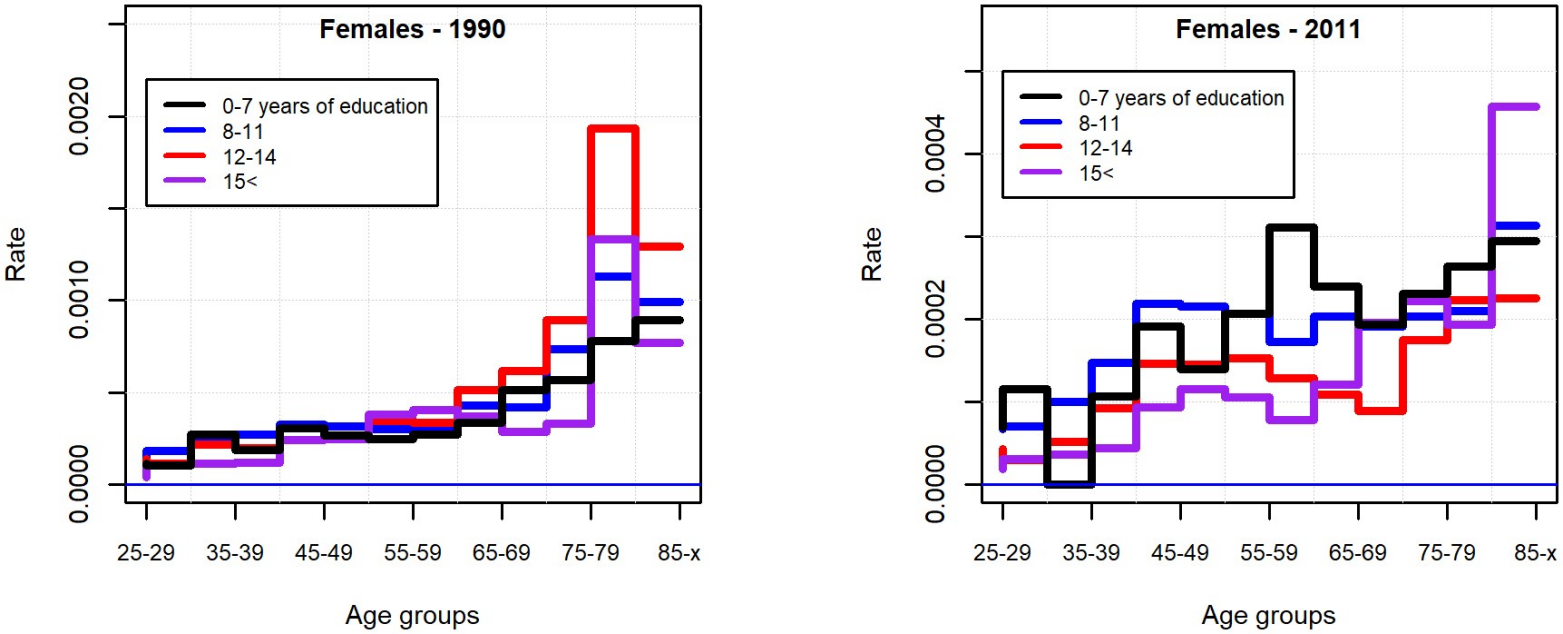
Suicide rate by educational attainment in 1990 and 2011 (females 25 years of age and above).

Men without secondary-school graduation at all ages committed more suicides in the beginning of the 90s and two decades later than their more educated peers (Fig 3). For women, the age-specific pattern of suicide rate by educational attainment was far from clear in 1990, while in 2011 the risk carried by those with low educational attainment was more pronounced (Fig 4).

**Fig 5:**
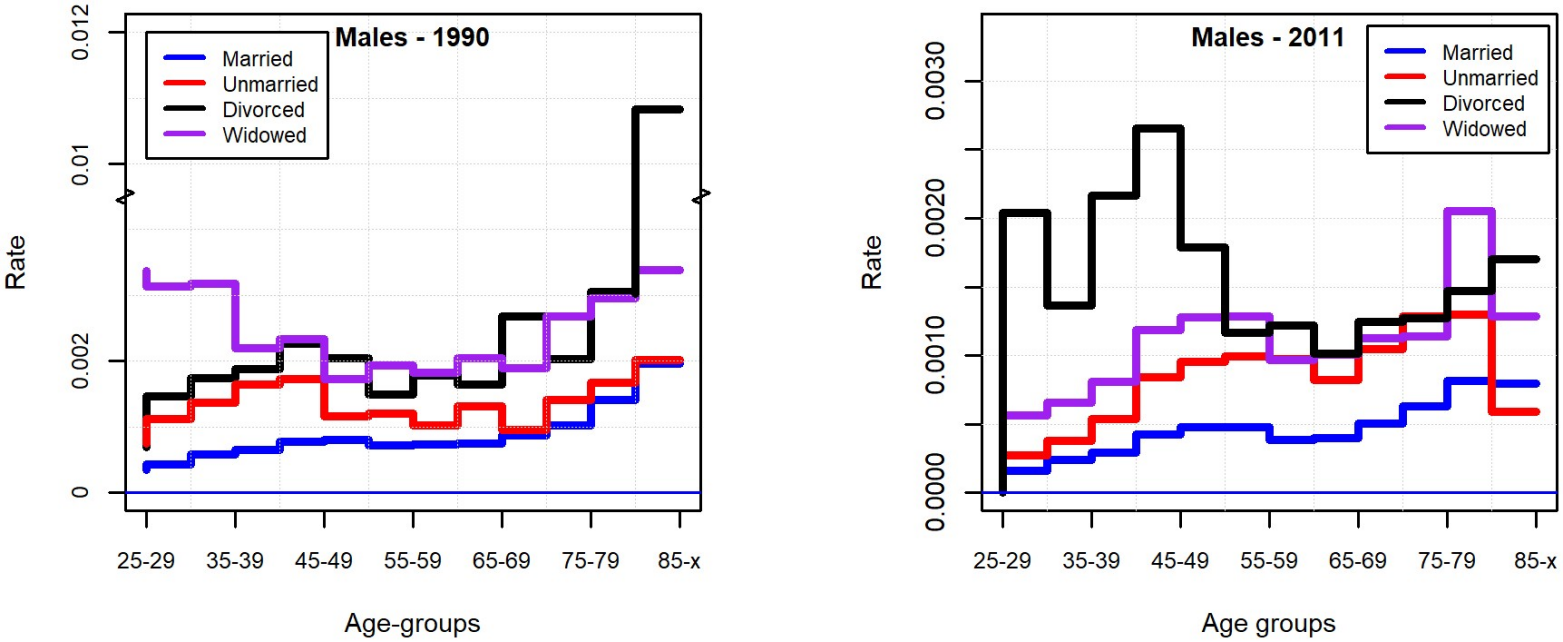
Suicide rate by marital status in 1990 and 2011 (males 25 years of age and above).

**Fig 6:**
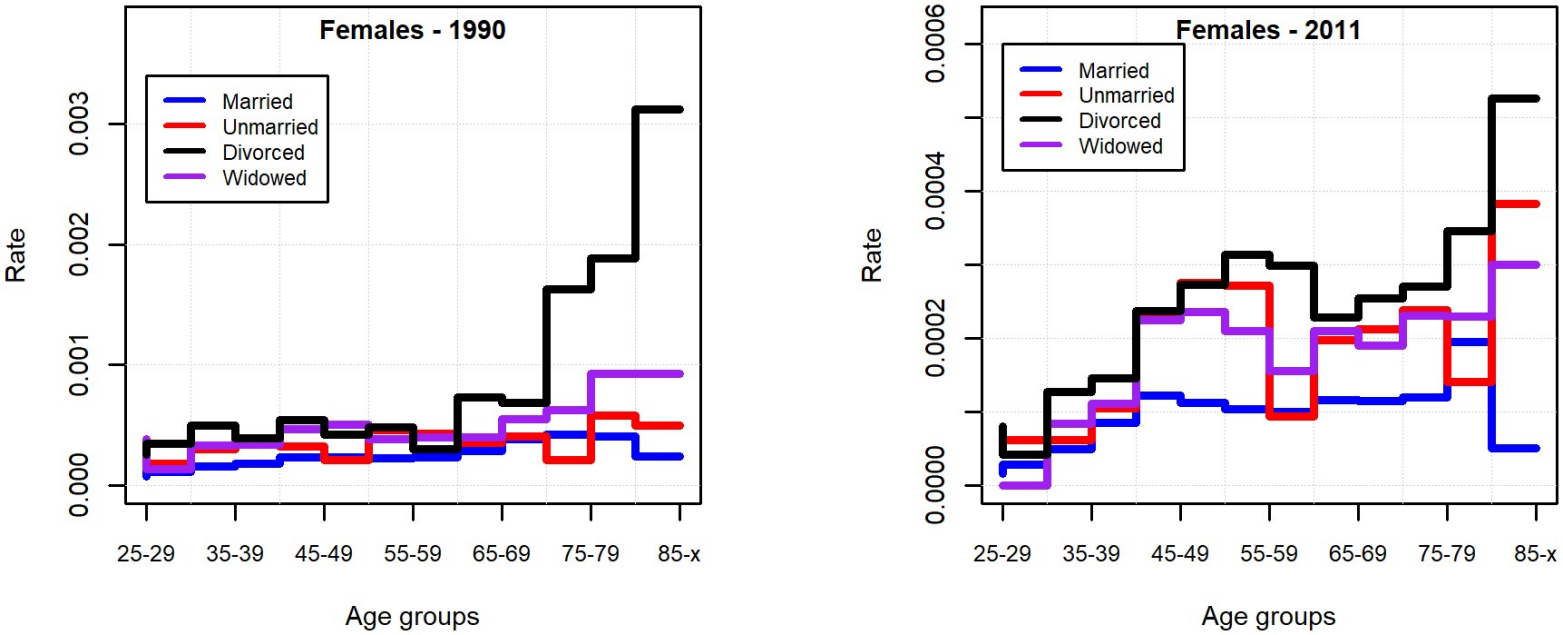
Suicide rate by marital status in 1990 and 2011 (females 25 years of age and above).

While for males the data for both 1990 and 2011 makes it obvious that the married group has the lowest suicide rate compared to any other marital status groups in the various age groups, for females this is obvious only for the data for 2011. Furthermore, it is easily perceivable that mostly the members of the divorced group carry the greatest risk (Figs 5 and 6).

### The results of the standardization and decomposition analysis (SDA)

The SDA analysis was carried out two times. The first time, it contained only the factors of educational attainment and age, but—for the whole population—a third factor (gender) was also included (Table 3). For the second time, the factor of marital status was included as well.

**Table 3:** The results of SDA by including the factors educational attainment and age.

Component effect	Standardization		Decomposition	
	Population 2011	Population 1990	Difference (Effect)	Percent Distribution of Effect
**Males**				
Age	71,00	67,90	3,11	-8,53
Education	63,54	75,36	-11,82	32,44
Adjusted rate effect / mean	56,21	83,94	-27,73	76,09
Crude rate / mean	53,07	89,51	-36,44	100,00
**Females**				
Age	24,84	21,48	3,37	-19,69
Education	22,05	24,28	-2,23	13,04
Adjusted rate effect / mean	14,62	32,85	-18,23	106,64
Crude rate / mean	13,71	30,80	-17,09	100,00
**Total**				
Age	45,94	42,98	2,96	-11,29
Education	41,30	47,45	-6,15	23,48
Gender	44,41	44,78	-0,37	1,41
Adjusted rate effect / mean	33,85	56,48	-22,63	86,40
Crude rate / mean	31,85	58,04	-26,19	100,00

The numbers in the last column of Table 3 show the percentage breakdown of the component effects, which expresses the relative contribution of the effect of individual components to the difference of raw rates. The sum of the percentages is 100.0%. A positive or a negative percentage indicates the proportion that the corresponding component effect contributes to or narrows the rate difference, respectively [38].

The results of the parsimonious SDA show that the improvement of educational level helped to decrease the rate for men by about a third, while for women the percentile contribution was much more modest (13.0%) which was counterbalanced solely by the change in the age composition (-19.7%) (Table 3). For men, however, the extent (-8.5%) of this latter effect was much smaller (Table 3). For men, in total a fourth of the change in suicide rate was linked to the change in population composition. Since the relation between educational attainment and suicide is much weaker for women, it is not surprising that the indirect effect of educational attainment was also smaller.

Among the sociodemographic variables, being not married is mostly a strong risk factor of suicide. Due to the increase in the number of people in the higher risk (i.e not married) marital statuses, marital status created an effect contrary to educational attainment: i.e. decreased the difference between the rates of the two periods (Table 4). For women, the percentage (-13.5%) surpassed the extent of the component effect occurring in educational level (10.1%), while the contribution of the age composition was even greater (-16.5%), which can be due to the higher proportion of the elderly (Table 4). For men, including the marital status resulted in component effects with similar direction but stronger than that for women. Whilst gender is in many regression models one if not the strongest predictors of suicide, interestingly enough no such thing was experienced in the SDA model: the change of gender composition had a negligible effect (2.7%) on the decrease of suicide rate compared to other factors (Table 4). By and large, compared to the component effects offsetting each other, the rate effect proved to be the most determining, which may refer to the role of the factors ignored during the decomposition, but can also indicate that these external effects affect each sociodemographic group and their age segments. The rate effect summarises these effects.

**Table 4:** The results of SDA by including the factors educational attainment, age and marital status.

Component effect	Standardization		Decomposition	
	Population 2011	Population 1990	Difference (Effect)	Percent Distribution of Effect
**Males**				
Age	73,51	69,47	4,04	-11,09
Education	65,74	77,28	-11,55	31,69
Marital status	76,10	67,09	9,01	-24,72
Adjusted rate effect / mean	53,65	91,59	-37,94	104,12
Crude rate / mean	53,07	89,51	-36,44	100,00
**Females**				
Age	24,85	22,02	2,83	-16,53
Education	22,58	24,31	-1,73	10,13
Marital status	24,70	22,39	2,30	-13,47
Adjusted rate effect / mean	13,94	34,42	-20,49	119,87
Crude rate / mean	13,71	30,80	-17,09	100,00
**Total**				
Age	47,22	43,94	3,28	-12,51
Education	42,57	48,44	-5,87	22,41
Marital status	48,52	42,85	5,67	-21,66
Gender	45,40	46,12	-0,71	2,71
Adjusted rate effect / mean	32,30	60,86	-28,56	109,05
Crude rate / mean	31,85	58,04	-26,19	100,00

## Discussion

Objective of the current study was to ascertain the contribution of changes in some sociodemographic factors to the decrease of the suicide rate between 1990 and 2010 in Hungary. According to our main results, the improvement of educational level helped to decrease the rate for men by about a third, while for women only by about a tenth. However, the benefit of the improvement in educational attainment during the period investigated was significantly offset by the changes primarily in marital status (the ratios of unmarried and divorced subjects increased for both genders) and in age distribution (the ratio of the elderly persons increased for both genders).

For long decades in the second half of the 20th century, Hungary had the highest suicide rate which went into a steep decline for women in the beginning of the 80s and in the second half of the decade for men too [1]. Starting at the end of the 80s, the financial and psychological burdens of the pervading political and economic transition interestingly enough did not moderate the improvement of the rate in Hungary contrary to the situation in many post-communist countries where the rate actually increased rather than decreased after the regime change [1, 9]. The explanations for the decrease are contradictory for the time being (see Introduction).

In our study, we searched for the answer to the question: What effect could the transformation of the demographic and socieconomic structure of the population have on the change of the suicide rate after the regime change? To answer this question, the standardization and decomposition analysis was used which is a widespread method in health sciences [34, 38, 39], while the two periods investigated represent important moments of the persistent decrease of the Hungarian suicide rate. The first (1989–91) is the period of the end of state-socialism, as well as the beginning of the lasting rate decrease, while the second (2010–12) represents a period two decades after the political transition. The results show that the improvement of educational attainment (i.e. the increase of the ratios of groups whose members graduated from secondary-school, college or university) contributed significantly to the decrease of male suicide rate, while this effect was much smaller for females. This much smaller effect of educational attainment for females is consistent with the results, which indicate gender differences in the effect of socioeconomic status on suicide [13, 21, 24]. The results of our study imply that the improvement of society’s educational attainment can be an important preventive measure against suicide at the population level (the possible factors mediating between educational attainment and suicidal behaviour were discussed in detail in the Introduction section). However, according to preliminary expectations, the changes in the composition of marital status counterbalanced these effects completely for women and partly for men.

The rate effect provoking the change indicates that behind the decrease of the suicide rate there must be factors which were capable of moderating the suicidal behaviour of all sociodemographic groups. The probability of this explanation is further reinforced by the fact that the decrease of suicide in individual Hungarian regions took place „proportionally”, and, accordingly, the spatial pattern of suicide did not change during this period [1, 4]. Similarly, the rate ratios between the highest and lowest educated males stayed practically unchanged. We believe that further investigations are needed to explore the reasons behind the decreasing rate of suicide.

This study has several limitations. First and foremost, we failed to account for important variables such as indicators of mental health care, alcohol consumption or rate of unemployment. Some of these may be important factors in the decrement of the Hungarian suicide rate after the political and economic transition. For instance, alcohol use is demonstrated as a risk factor for suicide at the levels of individuals [40]. Furthermore, with some exceptions [e.g. 12], the majority of studies have found that per capita alcohol consumption (or other measures of drinking habits) and suicide rate are associated positively at the population level [35, 41–43]. Using the proxy variables pure alcohol consumption per capita and the number of deaths due to liver cirrhosis to assess the number of subjects with alcohol use problems, we may say with great certainty that in Hungary the number of these subjects decreased during the period investigated here (1990–2011) [36, 44, 45]. Another possible limitation of our study, as we used “unlinked data”, is the so-called “numerator-denominator bias” [13, 46]. Furthermore, it is also possible that the factors provoking the decrease of the rate differed from period to period, as well as in terms of their magnitude/significance. We are aware that psychiatric disorders, especially mood disorders and also their subsyndromal manifestations affective temperaments are among the most important risk factors for suicide [47–50]. Unfortunately, we had no data on the psychiatric status of the individuals in our sample which should also be considered as a limitation of the study.

## Supporting information

S1 Data (RAR)
